# Report of a Case of Ulceration of the Larynx, Perichondritis of the Arytenoid Cartilages, Abscess and Partial Exfoliation of Both Cartilages Resulting from Typhoid Fever

**Published:** 1913-09

**Authors:** J. H. Bryan

**Affiliations:** Washington


### Report of a Case of Ulceration of the Larynx, Perichondritis of the Arytenoid Cartilages, Abscess and Partial Exfoliation of Both Cartilages Resulting From Typhoid Fever.

This case is reported in order to emphasize the importance of making regular and systematic examinations of the upper air passages, especially of the larynx, in all cases of typhoid fever, in order to detect the early changes that take place in the mucous membrane of the upper air passages in this disease. The frequency of this complication in typhoid fever in Europe, according to Landgraf, is 11 per cent of all fatal cases; according to Griesinger, 26 per cent; Kanthack, 26 per cent; Ouskow, 30 per cent. It is difficult to arrive at any conclusion as to the comparative frequency of this complication of typhoid fever in this country and abroad. The figures given by Jackson seem to show that a much larger number of cases of laryngeal involvement occur in this country than is indicated by the figures given by Thompson. The epidemic in which Jackson made his observations was, however, an unusually severe one, and the subjects were largely of a poorly nourished type, and this may account for the apparently greater frequency of this complication in this country. We cannot get at the truth in this matter until more careful observations are made, not only in the hospitals, but in private practice as well.—Dr. J. H. Bryan, Washington.

